# YOLOv11-GLIDE: An Improved YOLOv11n Student Behavior Detection Algorithm Based on Scale-Based Dynamic Loss and Channel Prior Convolutional Attention

**DOI:** 10.3390/s25226972

**Published:** 2025-11-14

**Authors:** Haiyan Wang, Guiyuan Gao, Wei Zhang, Kejing Li, Na Che, Caihua Yan, Liu Wang

**Affiliations:** College of Computer Science and Technology, Changchun University, Changchun 130022, China; gaoguiy0409@163.com (G.G.); wzhang@sina.cn (W.Z.); likejing1011@163.com (K.L.); chen@ccu.edu.cn (N.C.); 241501504@ccu.edu.cn (C.Y.)

**Keywords:** YOLOv11n, Scale-based Dynamic Loss, Channel Prior Convolutional Attention, Sparse Depthwise Convolution

## Abstract

Student classroom behavior recognition is a core research direction in intelligent education systems. Real-time analysis of students’ learning states and behavioral features through classroom monitoring provides quantitative support for teaching evaluation, classroom management, and personalized instruction, offering significant value for data-driven educational decision-making. To address the issues of low detection accuracy and severe occlusion in classroom behavior detection, this article proposes an improved YOLOv11n-based algorithm named YOLOv11-GLIDE. The model introduces a Channel Prior Convolutional Attention (CPCA) mechanism to integrate global and local feature information, enhancing feature extraction and detection performance. A scale-based dynamic loss (SD Loss) is designed to adaptively adjust the loss weights according to object scale, improving regression stability and detection accuracy. In addition, Sparse Depthwise Convolution (SPD-Conv) replaces traditional down-sampling to reduce fine-grained feature loss and computational cost. Experimental results on the SCB-Dataset3 demonstrate that YOLOv11-GLIDE achieves an excellent balance between accuracy and lightweight design. Compared with the baseline YOLOv11n, mAP@0.5 and mAP@0.5-0.95 increase by 2.5% and 7.6%, while Parameters and GFLOPS are reduced by 9.4% and 11.1%, respectively. The detection speed reaches 127.9 FPS, meeting the practical requirements of embedded classroom monitoring systems for accurate and efficient student behavior recognition.

## 1. Introduction

With the rapid development of the intelligent education system, real-time recognition of classroom behavior has become an important research direction to understand students’ learning participation and improve teaching quality. Effective classroom behavior recognition can provide a quantitative basis for students’ attention analysis, teaching evaluation, and personalized guidance, which is of great significance for realizing educational data-driven decision-making. However, the classroom scene has the characteristics of dense groups of students, frequent occlusion, and a small action range, which brings great challenges to high-precision and stable behavior detection. Therefore, it is of great significance to study a high-precision, lightweight, and robust student behavior detection algorithm to promote the intelligent development of a smart education system. This kind of algorithm can not only help teachers accurately grasp the classroom dynamics and realize personalized teaching and differentiated management, but also provide technical support for automated classroom analysis and teaching quality evaluation in smart education.

In the field of classroom behavior detection, YOLO series models have become the core direction. In recent years, more and more research work has been conducted in the field of classroom student behavior detection. Domestic research focuses on engineering implementation. Sheng et al. [1] proposed an improved YOLOv8s-based real-time detection method, designing a multi-scale large kernel convolution module (MLKCM) and a progressive feature optimization module (PFOM); through multi-axis pooling, it achieves adaptive receptive fields and segmented channel dimensions to aggregate local–global information, yet it overemphasizes feature extraction optimization and fails to fully resolve fine-grained information loss in small-scale behaviors. Additionally, Zhang et al.’s PLA-YOLOv11n [2] integrates partial convolution (PConv) [3] into the C3K2 module to build the C3K2-PConv structure, embeds a large kernel self-attention (LSKA) [4] mechanism, replaces SPPF with an attention feature integration module (AIFI) [5], and adds a high-resolution P2 detection head; this model significantly enhances occluded scene robustness and small-object detection ability, but still has room for parameter control optimization and fails to address training loss fluctuations caused by label box subjective errors. Other domestic efforts include compressing YOLO parameters to less than 30% of the original via lightweight backbones; for example, researchers adopt lightweight networks like MobileNetV3 [6] and ShuffleNet [7] to achieve this compression, which helps the model adapt to embedded devices. Another direction is alleviating student occlusion-induced detection deviation; this is performed by optimizing the anchor box generation strategy of the region proposal network (RPN) [8] and combining it with the feature pyramid network (FPN) [9] to optimize multi-scale feature fusion. Some teams also explore multi-modal fusion of YOLO visual detection with classroom audio and infrared thermal imaging data; a specific case is aligning the peak value of speech energy with the spatiotemporal coordinates of the “standing” behavior, which improves the timeliness of group abnormal behavior detection to the second level. However, most domestic improvements focus on a single dimension; for instance, some only optimize the model in terms of lightweight design, while others only address the problem of occlusion, and they all ignore the key pain point of fine-grained feature loss.

By contrast, international research emphasizes basic theoretical innovation and interdisciplinary integration. Some studies combine the latest YOLO versions with advanced architectures; specifically, they introduce the Transformer [10] architecture, and also adopt group behavior modeling based on graph neural networks (GNN) [11]. Through graph convolutional networks (GCN) [12], these studies abstract student–teacher interactions into dynamic graph structures, and combine them with the behavior coordinate features detected by YOLO to analyze the correlation between seat adjacency and behavior states—this enables early warning of group anomalies 15–30 s in advance. Other studies integrate educational psychology theories to construct a “behavior–cognition” correlation model; they combine the detection results of YOLO with the evaluation of students’ learning status, which provides an educational explanatory basis for the detection outcomes. Nevertheless, these international methods—especially those based on Transformer or GNN—often suffer from a surge in computation, making it hard to balance accuracy and embedded deployment requirements.

Aiming to address the problems of low detection accuracy, unstable model, and information loss in the above-outlined task of classroom behavior detection, this article innovates from three aspects: feature extraction optimization, loss function stability improvement, and structural lightweight design when improving YOLOv11. The main contributions of this article are as follows:

(1) The original feature extraction module of YOLOv11n has the problem of insufficient capture of local and global information when dealing with complex occlusion and multi-scale behavior, which makes it difficult for the model to accurately focus on key areas. To this end, this article introduces the CPCA [13] to achieve adaptive enhancement of the salient target area by fusing the two-dimensional feature attention of the channel and space, thereby improving the accuracy and robustness of feature extraction.
(2) The traditional loss function is susceptible to labeling errors and IoU jitter in small target detection, resulting in unstable training. In order to solve this problem, a scale-based dynamic loss function, SD Loss [14], is proposed, which adaptively adjusts the weight ratio of scale and position loss according to the actual area of the target, which significantly improves the detection stability for small-scale behavior.
(3) In the embedded deployment environment, the step convolution of YOLOv11n can easily cause fine-grained information loss and increase the computational burden. In this article, the sparse deep convolution (SPD-Conv) [15] module is used to replace the traditional down-sampling method, which can effectively retain high-resolution features while reducing the number of parameters and calculations, so that the model still has strong feature expression ability under lightweight conditions.

## 2. YOLO Series

### 2.1. The Evolution of YOLO Architecture

As the core technology in the field of real-time object detection, the continuous iteration of the YOLO series provides key support for classroom behavior detection and becomes the mainstream baseline model in this scenario [16]. YOLOv1 [17] pioneered the single-stage detection idea in 2016, transforming the detection task into an end-to-end regression problem, realizing the initial balance between ‘real-time’ and ‘detection accuracy’ for the first time. YOLOv2 [18] introduces an anchor frame and dimension clustering strategy to optimize target positioning accuracy, and improves training stability through batch normalization. YOLOv3 [19] innovatively proposes a feature pyramid network to achieve multi-scale target detection and also derives a lightweight version, YOLOv3-tiny [20]; it simplifies the backbone network to reduce nearly 80% of parameters while maintaining basic real-time detection performance. Subsequent versions are continuously optimized, and YOLOv4 [21] integrates multiple technologies to enhance the robustness of complex scenes. YOLOv5 [22] designs a multi-version model to improve engineering adaptability. YOLOv6 [23] adopts CSPStackRep in the backbone to balance feature extraction efficiency and computational cost, uses Rep-PAN in the neck to strengthen cross-scale feature fusion, and introduces a decoupled head to accelerate the convergence of classification and regression tasks; YOLOv8 [24] unifies multi-tasking and uses double decoupling heads to improve adaptability; and YOLOv10 [25] adopts an improved anchor-free design to reduce dependence on preset anchors, integrates detection and segmentation capabilities into a single framework, and applies model compression technology to reduce memory usage. YOLOv11 [26] follows the CSPDarknet backbone network and introduces a new module to improve the efficiency of feature extraction. At the same time, it optimizes the neck network and the prediction end. Its ‘precision–speed–lightweight’ balance makes it an ideal baseline for classroom behavior detection. On this basis, YOLOv12 [27] further introduces an Area Attention and Residual Efficient Layer Aggregation Network (R-ELAN), which enhances the global feature interaction ability while maintaining real-time detection performance, but still fails to solve the core problems in the classroom scene.

### 2.2. YOLOv11n

**Figure 1:**
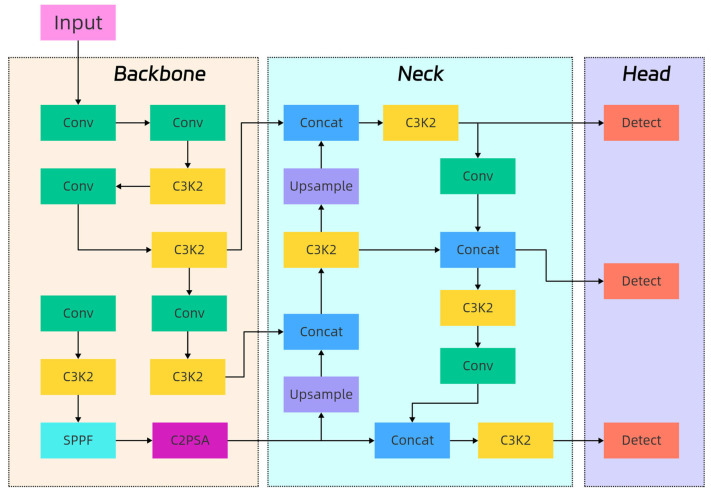
YOLOv11n network structure.

In the field of target detection, YOLO series algorithms have attracted much attention due to their high accuracy and efficiency. As a representative of the new generation, YOLOv11n has been improved and upgraded on the basis of previous generations. Its network structure has been optimized, and its feature extraction ability, detection speed, and accuracy have been improved to a certain extent. The YOLOv11n model has a smaller scale and lower computational complexity and is more easily deployed in the classroom monitoring terminal. It can maintain high-precision detection performance under limited computing power conditions and meet the comprehensive needs of educational intelligence systems. Therefore, this study chooses YOLOv11n as the baseline for improvement. It provides a possible auxiliary tool for the intelligent development of education, which helps teachers reduce part of the burden of teaching management and improve the teaching effect. The network structure of YOLOv11n is shown in Figure 1.

The key role of the YOLOv11n backbone feature network is to extract multi-level feature maps of the input image. These feature maps can capture image information of different scales and complexities, laying the foundation for subsequent object detection. In the backbone network, YOLOv11n follows the CSPDarkNet network structure of previous generations and introduces a new C3k2 mechanism to replace the C2f structure of YOLOv8. C3k2 improves the efficiency of feature extraction through two parallel convolution modules and a residual connection. At the same time, the backbone network retains the SPPF module and adds a C2PSA module with an attention mechanism. With the help of multiple PSA modules, the feature extraction ability is enhanced to better adapt to the complex and changeable data situation.

The neck network is responsible for fusing the feature information extracted by the backbone network and further performing feature extraction. YOLOv11n uses the C3k2 module to replace the traditional C3 [28] module in the neck network and uses the PAN-FPN [29] structure, as did the previous-generation YOLO algorithm. By up-sampling and down-sampling the feature maps of different sizes and then fusing them, the deep image features are extracted and a large amount of shallow semantic information is retained, so as to improve the accuracy of the network.

The head network completes the object location and classification according to the fused feature map. It can accurately identify the position of the bolt in the image and determine its state, such as whether it is loose or falling off. Differently from the single detection head of YOLOv5, YOLOv11n draws on the design of YOLOv8 and uses two decoupling heads, responsible for classification and detection tasks, respectively. In addition, YOLOv11n adds two deep separable convolutional layers to the classification detection head. Compared with the ordinary convolutional layer of YOLOv8, this convolutional layer performs convolution processing on each input channel separately and then fuses channel information through 1 × 1 point convolution, which significantly reduces the amount of calculation and improves the running speed of the network.

YOLOv11n not only has significant real-time and accuracy advantages but will also continue to make breakthroughs in detection accuracy, small target recognition, and occlusion processing, providing more powerful technical support for the development of intelligent education.

## 3. YOLOv11-GLIDE Architecture

YOLOv11n has achieved a good balance in detection accuracy, inference speed, and a lightweight model, but there are still some performance limitations in complex classroom scenarios. In the classroom environment, students have various postures, significant differences in action amplitude, and are often accompanied by occlusion, resulting in insufficient performance of the model in fine-grained feature extraction and small target detection. At the same time, the loss function with fixed weight lacks an adaptive adjustment mechanism in the face of different scale targets, and is highly sensitive to the labeling error of small targets, thus affecting the training stability. In addition, YOLOv11n still has the problem of feature information loss during the down-sampling process, which limits the model’s ability to characterize high-resolution details. In view of the above-mentioned problems, this article proposes an improved student behavior detection YOLOv11-GLIDE model based on YOLOv11n. The model introduces CPCA to enhance the global and local information interaction in the feature extraction process, uses SD Loss to improve the regression stability of targets at different scales, and uses SPD-Conv instead of traditional step convolution, thereby reducing feature information loss and computational overhead. The experimental results show that the model significantly improves the detection accuracy and robustness in complex classroom scenarios while remaining lightweight and provides an efficient and stable solution for real-time student behavior recognition in a smart education environment.

### 3.1. Channel Prior Convolutional Attention

The distribution of the single weight of the traditional attention mechanism will make the noise limit the classification performance of the network. This article introduces CPCA. This mechanism enhances the recognition ability of the model to features by optimizing the feature extraction process, thereby improving the accuracy of detection.

The CPCA includes two core modules: channel attention and spatial attention. The channel attention module strengthens the key features by analyzing and weighting the importance of each channel in the feature map; the spatial attention module mines spatial key information to provide a basis for the former and collaboratively optimizes the feature map. The channel prior convolution attention considers that the joint attention of the channel and the spatial dimension helps to capture the object features more accurately, and solves the problem of insufficient adaptive ability of the existing attention mechanism in dealing with complex scene object detection.

**Figure 2:**
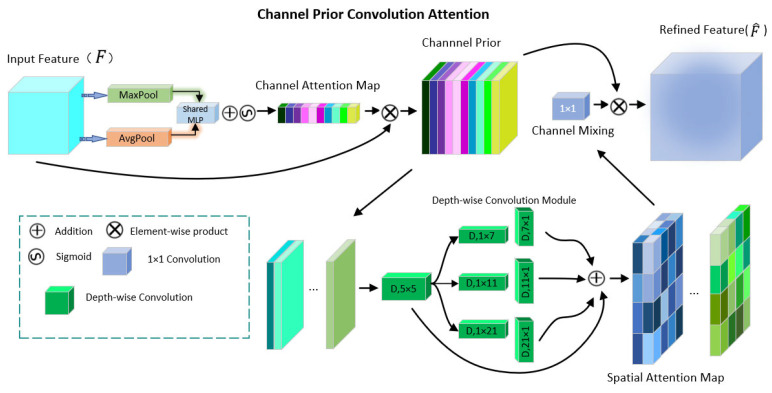
CPCA network structure.

The channel prior convolution attention mechanism is shown in Figure 2.

The CPCA first uses average pooling and maximum pooling operations to integrate spatial information from feature mapping and then passes it into a common multi-layer perceptron (MLP). Deep separable convolution is used to capture the spatial relationship between features, which ensures the retention of the relationship between channels and reduces the computational complexity. A multi-scale structure is used to enhance the ability of the convolution operation to capture spatial relationships. Finally, a 1 × 1 convolution is used for channel mixing. The CPCA process formula is

(1) $$\hat{F}=SA\left[CA\left(F\right)⊗F\right]⊗\left[CA\left(F\right)⊗F\right]$$

Among them, $\hat{F}$ represents the final output feature, CA represents the channel attention module, and SA represents the spatial attention module.

The channel attention expressions of CPCA are as follows:

(2) $$CA\left(F\right)=sigmoid\left\{MLP\left[AvgPool\left(F\right)\right]+MLP\left[MaxPool\left(F\right)\right]\right\}$$

The spatial attention part uses deep separable convolution to extract the spatial relationship between features, which retains the relationship between channels and reduces the computational complexity. The multi-scale structure adopted can effectively enhance the extraction of spatial relationships by convolution operations, calculated as follows:

(3) $$SA\left(F\right)={Conv}_{1\times 1}\left\{\sum_{i=0}^{3}{Branch}_{i}\left[Dw_{Conv\left(F\right)}\right]\right\}$$

Here, Dw_Conv represents a depth separable convolution and ${Branch}_{i}$ represents the i-th branch.

The CPCA uses channel statistics to generate channel prior weights, and then maps them into spatial position-sensitive weights through learnable convolution to realize the joint enhancement of channel semantics and spatial context, so that the model can accurately focus on key channels and salient spatial regions in complex backgrounds.

### 3.2. Scale-Based Dynamic Loss

SD Loss is proposed to solve the shortcomings of the existing loss function in small object detection. In small object detection, the loss (Sloss) based on the intersection over union (IoU) fluctuates greatly, especially for small objects, which will have a negative impact on model stability and regression. For mask labels, the fuzzy boundary will lead to Sloss fluctuation, and the instability of small objects is more obvious. It is also difficult for the position loss (Lloss) to converge when detecting missing objects, and it is easy to produce more false positives.

SD Loss dynamically adjusts the influence coefficients of Sloss and Lloss based on the object scale to reduce the impact of inaccurate labels on the stability of the loss function. For the bounding box (BBox) label, the small object’s Sloss is assigned a lower attention weight; for mask labels, the influence of Sloss is enhanced to ensure that the model pays more attention to Sloss.

$L_{BS}$ represents the scale loss based on the BBox and $L_{BL}$ indicates position loss based on bounding boxes, calculated as follows:

(4) $$L_{BS}=1-IoU+\alpha v$$

(5) $$L_{BL}=\frac{\rho^{2}\left(b_{p},b_{gt}\right)}{c^{2}}$$

Among them, $L_{BS}$ is used to measure the difference in scale between the predicted bounding box and the real bounding box, $L_{BL}$ is used to measure the difference in position between the predicted bounding box and the real bounding box, and IoU stands for Intersection over Union, which is the ratio of the intersection area to the union area of the predicted bounding box and the real bounding box. It is an important index to measure the overlap degree of the two bounding boxes. The value range is between 0 and 1, and the larger the value is, the higher the overlap degree of the two bounding boxes is. α is the weight coefficient, is used to balance the IoU loss and the influence of v, and v is used to measure the consistency of the aspect ratio of the bounding box, which reflects the difference between the predicted bounding box and the real bounding box in the aspect ratio. The ρ Euclidean distance function is used to calculate the Euclidean distance between two points.

$b_{p}$ predicts the centroid coordinates of the bounding box, the centroid coordinates of the real boundary frame of $b_{gt}$, and c represents the diagonal length of the minimum circumscribed rectangle of the two bounding boxes (predicted bounding box and real bounding box).

The scale loss $L_{MS}$ and position loss $L_{ML}$ of the mask are calculated as follows:

(6) $$L_{Ms}=1-\omega \frac{{|M}_{p}\cap M_{gt}|}{\left|M_{p}\cup M_{gt}\right|}$$

(7) $$L_{ML}=1-\frac{min\left(d_{p},d_{gt}\right)}{max\left(d_{p},d_{gt}\right)}+\frac{4}{\pi^{2}}{\left(\theta_{p}-\theta_{gt}\right)}^{2}$$

Among them, $M_{p}$ represents the predicted object pixel set, that is, the pixel set of the object area predicted by the model; $M_{gt}$ represents the real object pixel set, that is, the pixel set of the actual labeled object area; and ω is used to describe the difference between the predicted pixel set $M_{p}$ and the real pixel set $M_{gt}$. It can adjust the calculation of $L_{MS}$ and play a balancing role. $d_{p}$ and $d_{gt}$ represent the distance from the average pixel of the predicted object to the origin in the polar coordinate and the distance from the average pixel of the real object to the origin in the polar coordinate, respectively. $\theta_{p}$ and $\theta_{gt}$ represent the angle of the average pixel of the predicted object under the polar coordinate and the angle of the average pixel of the real object under the polar coordinate, respectively.

When the model scales the image or samples the feature map twice, the size of the object will change. In order to determine the true object size, the ratio of the object scale to the original image and the current feature map (ROC) needs to be calculated.

(8) $$R_{OC}=\frac{w_{O}\times h_{O}}{w_{C}\times h_{C}}$$

Here, $w_{O}$ and $h_{O}$ are the width and height of the original image and $w_{C}$ and $h_{C}$ are the width and height of the current feature map.

The influence coefficients $\beta_{B}$ and $\beta_{M}$ of scale loss and position loss are calculated as follows:

(9) $$\beta_{B}=\min\left(\frac{B_{gt}}{B_{gt\;max}}\times R_{OC}\times \delta ,\delta \right)$$

(10) $$\beta_{M}=\min\left(\frac{M_{gt}}{M_{gt\;max}}\times R_{OC}\times \delta ,\delta \right)$$

Among them, $B_{gt}$ is the area of the current object box and $B_{gt\;max}$ is the maximum area of the small object, which defaults to 81; $M_{gt}$ refers to the area of the real mask area of the object, which represents the number of pixels actually occupied by the object in the image; and $M_{gt\;max}$ is a preset maximum object mask area, which is defined as 81 by the International Association of Photoelectric Instrument Engineers to normalize the object area. δ is an adjustable hyperparameter.

The scale-based dynamic bounding box loss (SDB loss) is calculated as follows:

(11) $$\beta_{L_{BS}}=1-\delta +\beta_{B}$$

(12) $$\beta_{L_{BL}}=1+\delta -\beta_{B}$$

(13) $$L_{SDB}=\beta_{L_{BS}}\times L_{BS}+\beta_{L_{BL}}\times L_{BL}$$

where $\beta_{L_{BS}}$ and $\beta_{L_{BL}}$ are the influence factors of $L_{BS}$ and $L_{BL}$, respectively. When the object BBox area is greater than 81, $L_{SDB}$ degenerates into CIoU loss. The scale-based dynamic mask loss (SDM loss) is calculated as follows:

(14) $$\beta_{L_{MS}}=1+\beta_{M}$$

(15) $${\;\beta }_{L_{ML}}=1-\beta_{M}$$

(16) $$L_{SDM}=\beta_{L_{MS}}\times L_{MS}+\beta_{L_{ML}}\times L_{ML}$$

where $\beta_{L_{MS}}$ and $\beta_{L_{ML}}$ are the influencing factors of $L_{MS}$ and $L_{ML}$, respectively.

Through this dynamic adjustment, SD Loss effectively improves the regression ability and detection performance of the neural network on different-scale objects. The existing object detection loss (CIoU, SLS, etc.) combines scale loss and position loss according to fixed weights, ignoring the objective phenomenon of ‘the smaller the frame/mask area, the greater the IoU jitter’ in the actual labeling, resulting in the network being overly dominated by noise IoU on small objects and overly tolerant of center point offset on large objects.

### 3.3. Sparse Depthwise Convolution

There are some common design flaws in the current convolutional neural network (CNN) [30] architecture, that is, the use of cross-line convolution or pooling layers, which will lead to the loss of fine-grained information and reduce the effectiveness of feature learning. In order to solve this problem, a new CNN module, SPD-Conv, is introduced. SPD-Conv is composed of a space-to-depth layer followed by a non-cross-line convolutional layer. It does not lose learnable information during the down-sampling process and retains all discriminant feature information as much as possible. It is suitable for most CNN architectures and can replace the cross-line convolution or pooling layer used by the original CNN architecture. It also has good performance in complex tasks with low image resolution and small objects. Among them, the SPD layer slices the intermediate feature map to generate multiple sub-feature maps and divides the sub-feature map based on the specific region of the original feature map. Each sub-feature map down-samples the original feature map through a specific scaling factor, thereby reducing the spatial dimension and increasing the channel dimension. Subsequently, a non-stepping convolutional layer is used to further process the features processed by the SPD layer, which reduces the number of channels through learnable parameters to reduce information redundancy while retaining key feature information.

**Figure 3:**
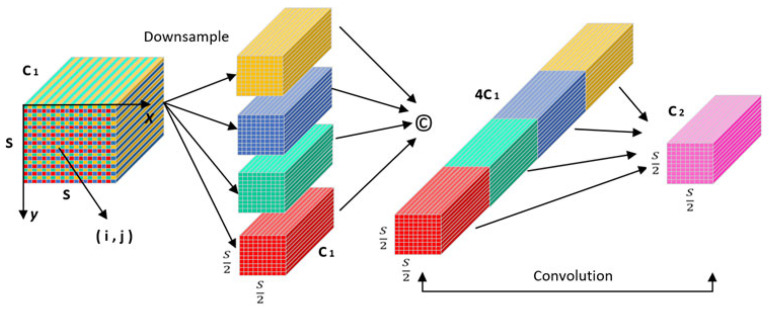
SPD-Conv network structure.

For the input feature map X with a size of $S\times S\times C_{1}$, the space-to-channel layer will first sample it at intervals to obtain ${scale}^{2}$ sub-feature maps with a size of $\frac{S}{scale}\times \frac{S}{scale}\times C_{1}$. Then, the feature map with the size of $\frac{S}{scale}\times \frac{S}{scale}\times {scale}^{2}C_{1}$ is obtained by channel splicing. The SPD-Conv structure is shown in Figure 3.

The sub-feature map obtained by sub-sampling the feature map X through the down-sampling parameter scale is expressed as follows:

(17) $$f_{0,0}=X\left(0:S:scale,0:S:scale\right)$$

(18) $$f_{0,scale-1}=X\left(0:S:scale,scale-1:S:scale\right)$$

(19) $$f_{scale-1,0}=X\left(scale-1:S:scale,0:S:scale\right)$$

(20) $$f_{scale-1,sacle-1}=X\left(scale-1:S:scale,scale-1:S:scale\right)$$

Here, 0:S:scale represents the elements from 0 to S, and one value is taken for each interval of scale elements.

The input image realizes structural reconstruction after passing through the space to the channel layer. The single-step 1 × 1 convolution layer can use the convolution kernel with a step size of 1 to perform cross-channel feature information interaction for the feature map after dimension transformation and adjust the number of channels. Therefore, for the small object recognition task, compared with the original step convolution feature extraction, this down-sampling method completely retains the feature information without worrying about redundancy.

### 3.4. Improved YOLOv11n Model Construction Based on Scale Dynamic Loss and Channel Prior Convolution Proof

**Figure 4:**
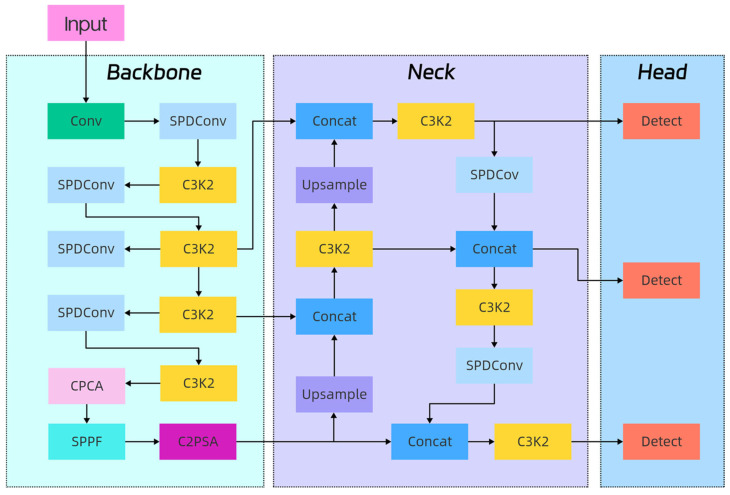
Improved YOLOv11-GLIDE network structure.

Combined with the above-outlined content, this article introduces the CPCA mechanism into the backbone part of the YOLOv11n model to dynamically allocate the attention weights of channels and spatial dimensions for the feature map, which effectively enhances the network’s feature extraction ability for the object and facilitates integration into the network. In order to better capture the detailed information of small objects and improve the detection ability of the model for small objects, the SPD-Conv convolution module is introduced in YOLOv11n to achieve effective feature learning. The SD Loss is migrated to the student classroom behavior detection scene, which can effectively alleviate the problems of small student action objects, subjective errors in the annotation box, and significant differences in different behavioral scales caused by long-distance camera shooting. When detecting small-amplitude actions such as reading and writing, SD Loss suppresses the training instability caused by the jitter of small object labels by dynamically reducing the IoU weight and improving the center point regression weight. When detecting large-amplitude actions such as raising hands and playing mobile phones, the inverse weighting is used to make full use of the scale information. Therefore, SD Loss is introduced in this article, and two losses are dynamically reweighted according to the real area of the object. The regression of small objects is more dependent on the position constraint, and the large objects are more dependent on the scale constraint, thus significantly weakening the influence of label error on training and improving the stability of cross-scale detection. The improved YOLOv11-GLIDE model is constructed, and the network structure is shown in Figure 4.

The YOLOv11-GLIDE model proposed in this study provides a feasible technical solution for the large-scale application of classroom behavior analysis. Its lightweight design supports real-time processing on edge computing devices. In practical applications, through the integration with the smart classroom system, it can provide teachers with real-time student participation statistics and behavior trend analysis, so as to support them in carrying out accurate teaching intervention and classroom rhythm adjustment, and promote teaching strategies from being experience-oriented to utilizing data-assisted decision-making. Although the YOLOv11-GLIDE algorithm proposed in this study has achieved a good balance between detection accuracy and efficiency, the application of AI technology to classroom behavior monitoring is still accompanied by potential risks and needs to be carefully evaluated. This mainly includes the potential violation of students’ privacy, the injustice of algorithm decision-making due to the deviation of training data, and the invisible pressure of continuous monitoring on students’ psychology. In order to promote the implementation of technology responsibly, it is recommended to follow the principles of data minimization and anonymization in deployment, ensure the transparency of the process and obtain informed consent, combine algorithm output with teachers’ humanistic judgment, and establish a multi-dimensional evaluation system to improve teaching efficiency while adhering to the ethical bottom line and avoiding the risk of technology abuse.

## 4. Experiments

### 4.1. Dataset

In this article, the open student classroom behavior dataset SCB-Dataset3 [31] is used as the experimental data source. In order to expand the scale of training samples and improve the adaptability of the model to diverse classroom scenarios, this study integrates its subsets SCB3-S and SCB3-U for model training and verification. Among them, 4215 images are selected as the training set, and 1057 images are used as the verification set. The division method of ‘training set + verification set’ is the best choice due to the single-application scenario, high labeling cost, and baseline comparison demand. The dataset contains a large number of student behavior samples in real classroom scenarios, covering six typical behaviors: raising hands, reading, writing, using mobile phones, bowing, and lying at desks. These data are collected by the classroom video surveillance system and strictly labeled and processed to ensure the accuracy and reliability of the samples, aiming to provide a unified experimental benchmark and data support for students’ classroom behavior detection tasks.

### 4.2. Experimental Environment

The training process of the model in this article is set to 200 iterations (epochs), and the step attenuation strategy is adopted. The initial learning rate (lr0) is set to 0.01, and the learning rate at the end of training is controlled by the final learning rate scale factor (lrf = 0.01). The final learning rate is calculated to be 0.01 × 0.01 = 0.0001, which not only ensures that the gradient update step size is sufficient in the early stage of training to converge quickly, but also reduces the step size in the later stage to stabilize the model performance. The computer configuration used for model training is shown in Table 1.

### 4.3. Evaluation Metrics

In order to comprehensively and objectively evaluate the comprehensive performance of the model in practical application scenarios, this article constructs an evaluation system from two core dimensions: performance effect and computational efficiency. Among them, the performance effect dimension takes precision, recall, and mean average precision (mAP) as the key indicators. Precision reflects the reliability of the prediction results, recall focuses on the target coverage ability of the model, and mAP comprehensively measures the overall performance of the model; the three together fully reflect the detection or classification ability of the model. In terms of computational efficiency, the computational complexity is measured by billions of floating-point operations per second (GFLOPS), the running speed is evaluated by the number of frames per second (FPS), and the degree to which the model is lightweight is measured by Parameters. Finally, through the collaborative analysis of multi-dimensional indicators, the system presents the comprehensive value of the model in practical applications. P, R, and mAP are calculated as follows:

(21) $$P=\frac{TP}{TP+FP}$$

(22) $$R=\frac{TP}{TP+FN}$$

(23) $$mAP=\frac{\sum_{k=1}^{n}PR}{N}$$

In the formula, P represents the accuracy rate, R represents the recall rate, TP represents the number of actual targets successfully detected by the model, FP represents the number of model error detection, FN represents the number of targets missed by the model, and N represents the number of categories contained in the sample dataset.

### 4.4. The Influence of Attention Mechanism on the Performance of Classroom Behavior Detection and Result Analysis

By designing the comparison experiment of the improvement effect of the attention mechanism, the influence of different attention mechanisms on the model detection effect is verified. Among them, the SE (Squeeze-and-Excitation) [32] channel attention mechanism can strengthen the feature channel through the compression and excitation process. The CBAM (Convolutional Block Attention Module) [33] combines channel attention and spatial attention, which can adaptively enhance the feature representation of key channels and spatial positions in the feature map, thereby improving the performance of the model. MSCA (Multi-scale Channel Attention) [34] uses a multi-branch dilated convolution structure to extract the context information of multi-scale receptive fields in parallel on the channel dimension and generates scale-aware channel weights through cross-branch interaction fusion, which adaptively strengthens the more discriminative channels in multi-scale features and improves the robustness of the model to scale-varying objects. EMA (Efficient Multi-scale Channel Attention) [35] efficiently captures cross-scale channel dependencies by grouping input features along the channel dimension and performing multi-scale one-dimensional convolution in parallel within each group, and achieves lightweight dynamic calibration of multi-scale channel weights without introducing additional parameters, thereby enhancing the ability to characterize key features at different scales. Through a one-dimensional convolution operation, ECA (Efficient Channel Attention) [36] can capture the correlation dependence between channels and reduce complex dimensionality reduction operations, thereby enhancing feature representation.

In the comparison experiment of the improvement effect of the attention mechanism, different attention mechanisms are introduced into the model, including SE, CBAM, MSCA, EMA, ECA, and CPCA, and the same parameter settings are used for training, so as to intuitively evaluate the performance of the models with different attention mechanisms. The experimental results are shown in Table 2, where ‘-’ denotes the YOLOv11n model without any attention mechanism.

Through experimental analysis, the YOLOv11n model increased mAP@0.5 by 0.3% after introducing the SE attention mechanism, increased mAP@0.5 by 0.6% after introducing the CBAM attention mechanism, increased mAP@0.5 by 0.9% after introducing the MSCA attention mechanism, increased mAP@0.5 by 0.1% after introducing the EMA attention mechanism, and increased mAP@0.5 by 1.5% after introducing the CPCA mechanism. The experimental results show that after introducing various attention mechanisms, the detection accuracy of the model has been improved to varying degrees, and the improvement effect of each index is the most obvious in the case of introducing CPCA. The comparison experiment of the attention mechanism verifies that CPCA has outstanding performance in improving the detection accuracy, which makes the model show stronger adaptability in complex scenes.

### 4.5. Performance Comparison and Result Analysis of Different Algorithms

**Figure 5:**
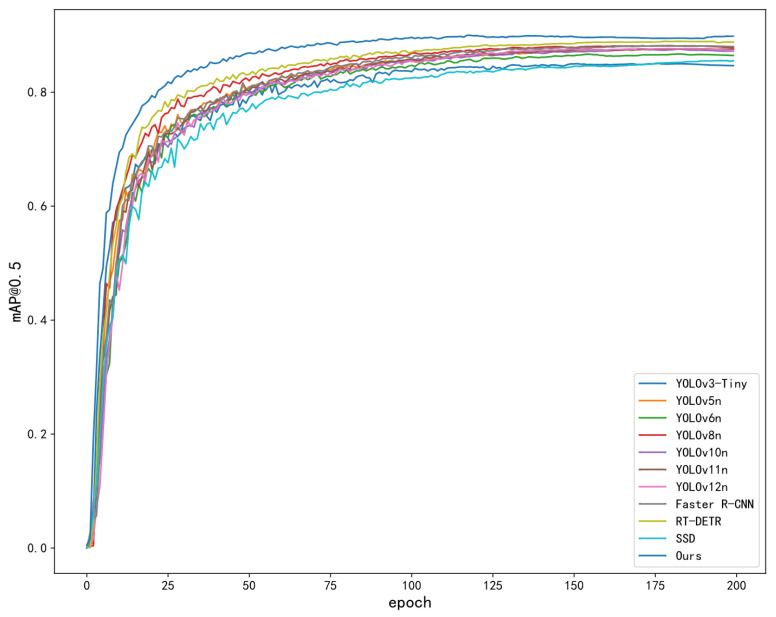
Comparison effect diagram.

In order to verify the performance of the YOLOv11-GLIDE model proposed in this article, a comparative experiment was designed. The improved YOLOv11-GLIDE model was compared with YOLOv3-tiny, YOLOv5n, YOLOv6n, YOLOv8n, YOLOv10n, YOLOv11n, and YOLOv12n models, as well as other representative object detection models like SSD [37], Faster R-CNN, and RT-DETR. By comparing the mAP and lightweight effects of each model, it can be seen that the effect of YOLOv11n is excellent, and it is selected as the baseline for improvement. The effect is shown in Table 3, indicating that it can intuitively evaluate the performance of the YOLOv11-GLIDE model. The mAP and lightweight effects are shown in Table 3 and Figure 5.

Through the analysis of the results, compared with the traditional YOLOv11n model, the mAP@0.5 of the YOLO-GLIDE model proposed in this article is increased by 2.5%, and the mAP@0.5-0.95 in the wider accuracy range is increased by 7.6%. Compared with YOLOv11n, YOLO-GLIDE’s higher precision means it makes fewer mistakes in object recognition. When it identifies something as a target object, that result is more likely to be correct, avoiding misjudging non-targets as targets. Meanwhile, its improved recall allows it to “see” more actual target objects in the scene. Compared to YOLOv11n, it misses fewer real objects that need to be detected. These two advantages together make YOLO-GLIDE more reliable in practical use, whether through reducing wrong identifications or ensuring that no key objects are overlooked. In addition to the detection accuracy, the computational complexity of the proposed YOLOv11-GLIDE model is also compared and analyzed. Although modules such as CPCA and SD Loss are introduced into the model, due to the lightweight SPD-Conv structure, the redundant feature extraction operation is effectively reduced, and the overall calculation amount is reduced. The experimental results show that compared with YOLOv11n, the model parameters and GFLOPS are reduced by 9.4% and 11.1%, respectively, and the inference speed reaches 127.9 FPS. When compared with other classic object detection models such as SSD, Faster R-CNN, and RT-DETR, SSD suffers from a relatively low mAP and a sluggish inference speed, while also having considerably higher model parameters and GFLOPS. Faster R-CNN manages to attain a respectable mAP@0.5 and recall. However, it comes with extremely high model parameters and GFLOPS, and its inference speed is remarkably slow, which renders it unfit for real-time applications. RT-DETR boasts a good mAP@0.5-0.95 and a decent inference speed. Yet, its model parameters and GFLOPS are still far higher than those of YOLOv11-GLIDE.

Compared with lightweight models such as YOLOv8n and YOLOv12n, YOLOv11-GLIDE achieves higher detection accuracy while maintaining lower computational cost, which proves that the proposed method achieves a good balance between accuracy and computational efficiency, and has the advantage of embedded real-time deployment.

To further verify the effectiveness and generalization ability of the model, we specifically selected the subset SCB3-U of the SCB-Dataset3 and conducted a comparative experiment with other recent relevant student behavior detection methods. The detailed experimental results are presented in Table 4.

As can be seen from Table 4, when compared with the performance of recent student behavior detection methods on the SCB3-U dataset, our model demonstrates distinct advantages. In detection accuracy aspects like precision, recall, and mAP@0.5, our model outperforms or is on par with other methods. Moreover, considering model parameter size, our model achieves a better balance between detection accuracy and model lightweightness. This shows that our model not only has strong student behavior detection capabilities but also has advantages in deployment on resource-constrained devices, fully verifying its effectiveness and superiority.

**Figure 6:**
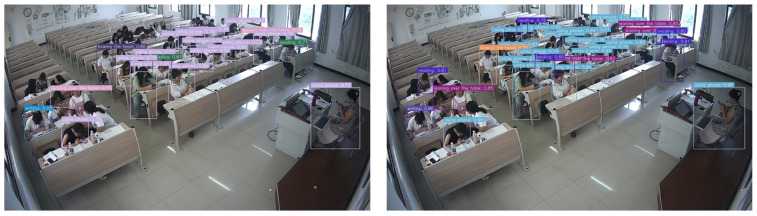
Comparison of recognition results between the baseline YOLOv11n (**left**) and the proposed YOLOv11-GLIDE (**right**).

The recognition effect before and after the improvement of the model is shown in Figure 6. The left side of Figure 6 is the test result of the initial YOLOv11n, and the right side is the test result of the YOLOv11-GLIDE proposed in this article. Comparing the left and right figures, it can be observed that the leakage of the original model is improved after improvement, the number of individual behaviors identified is increased, and the confidence is relatively high, which fully verifies the effectiveness and advancement of the proposed method in complex scenes.

In conclusion, experiments verify that the YOLOv11-GLIDE model has excellent performance in detection accuracy and shows significant advantages in computational complexity and model lightweightness, which proves that the YOLOv11-GLIDE model proposed in this article has considerable practicability and effectiveness in resource-constrained environments.

### 4.6. Ablation Experiment and Result Analysis

In order to verify the improvement effect of the three improvement points on the performance of the model, a series of ablation experiments were designed. The CPCA mechanism, SPD-Conv module, and the SD Loss are combined with YOLOv11n for experimental comparison. In this way, the specific improvement effect of each combination on the performance of the model can be clearly observed and compared. The results of ablation experiments are shown in Table 5.

Through the analysis of the results, after introducing CPCA into the YOLOv11n model, the mAP, precision, and recall of the model were significantly improved, while the GFLOPS and parameters of the model did not increase significantly. After introducing the SD Loss, the model is more stable in bounding box regression, thus effectively improving the detection accuracy of the model. In addition, after the SPD-Conv module is introduced into the model, the detection accuracy of the model is improved while the parameters and GFLOPS are significantly reduced. Therefore, ablation experiments verify that the model combined with three optimization strategies significantly reduces the computational complexity and resource consumption of the model, and effectively improves the detection accuracy of the model.

## 5. Conclusions

Aiming to address the problems of low detection accuracy, small target instability, and high computational complexity in the actual classroom environment, this study proposes an improved student classroom behavior detection algorithm: YOLOv11-GLIDE. The algorithm has three innovations on the basis of YOLOv11n. Firstly, the channel prior convolution attention mechanism (CPCA) is introduced to effectively integrate global and local context information, which significantly improves the model’s ability to focus on salient behavior regions in complex background and occlusion scenes. Secondly, a scale-based dynamic loss function (SD Loss) is designed, which can adaptively adjust the balance between scale loss and position loss according to the target scale, so as to improve the regression stability and detection accuracy of different scale targets. Finally, sparse deep convolution (SPD-Conv) is used to replace the traditional down-sampling operation, which reduces the number of parameters and calculation while retaining fine-grained feature information to ensure the efficient and lightweight performance of the model. The experimental results on the public SCB-Dataset3 dataset show that YOLOv11-GLIDE achieves significant improvement in detection performance and computational efficiency compared with the baseline model YOLOv11n; mAP@0.5 and mAP@0.5-0.95 are increased by 2.5% and 7.6%, respectively, and the number of parameters and the amount of calculation are reduced by 9.4% and 11.1%, respectively. At the same time, the model detection speed reaches 127.9 FPS, which realizes the organic unity of high precision and real-time performance. Overall, YOLOv11-GLIDE achieves an excellent balance between accuracy, robustness, and lightweight design, and provides an efficient and scalable technical solution for real-time behavior recognition and teaching behavior analysis in intelligent classroom scenarios.

## Figures and Tables

**Table 1:** Experimental environment.

Environment Configuration	Name	Related Configuration
Hardware environment	CPU	12v CPU Intel(R) Xeon(R) Platinum 8352 V CPU @ 2.10 GHz
	Running memory	24 GB
	GPU	NVIDIA GeForce RTX 4090
Software environment	Operating system	Ubuntu 22.04
	Python	3.12
	PyTorch	2.3.0
	CUDA	12.1

**Table 2:** Comparison of improvement effects of attention mechanism.

Model	Precision	Recall	mAP@0.5	mAP@0.5-0.95	Parameters/10^6^	GFLOPS	FPS (Frame/s)
-	0.859	0.832	0.876	0.673	2.6	6.3	126.6
SE	0.862	0.832	0.879	0.669	2.6	6.3	125.8
CBAM	0.864	0.833	0.881	0.672	2.6	6.4	124.2
MSCA	0.868	0.836	0.884	0.682	2.7	6.5	123.9
EMA	0.866	0.831	0.883	0.675	2.6	6.5	124.8
ECA	0.861	0.832	0.877	0.667	2.6	6.3	126.1
CPCA	0.871	0.838	0.889	0.702	2.7	6.5	123.7

**Table 3:** Comparison of detection performance and lightweight effect.

Model	Precision	Recall	mAP@0.5	mAP@0.5-0.95	Parameters/10^6^	GFLOPS	FPS (Frame/s)
YOLOv3-tiny	0.819	0.804	0.827	0.597	12.1	18.9	112.1
YOLOv5n	0.833	0.821	0.865	0.657	2.5	7.1	117.4
YOLOv6n	0.847	0.826	0.857	0.656	4.2	11.8	121.4
YOLOv8n	0.851	0.830	0.872	0.673	3.0	8.1	125.5
YOLOv10n	0.853	0.819	0.875	0.674	2.7	8.2	114.3
YOLOv11n	0.859	0.832	0.876	0.673	2.6	6.3	126.6
YOLOv12n	0.856	0.828	0.877	0.676	2.6	6.3	123.2
SSD	0.848	0.794	0.855	0.614	17.8	12.3	87.4
Faster R-CNN	0.867	0.837	0.882	0.628	47.5	71.8	8.8
RT-DETR	0.869	0.840	0.889	0.689	44.7	27.6	115.2
Ours	0.879	0.842	0.898	0.724	2.3	5.6	127.9

**Table 4:** Comparison of detection results of different algorithms on SCB3-U dataset.

Model	Precision	Recall	mAP@0.5	mAP@0.5-0.95	Parameters/10^6^
CSB-YOLO [38]	0.855	0.824	0.747	0.576	1.9
SBD-Net [39]	0.863	0.816	0.745	0.577	36.5
PLA-YOLO11n	0.871	0.824	0.764	0.513	18.4
Ours	0.879	0.842	0.776	0.582	2.7

**Table 5:** Ablation experimental results.

Model	Precision	Recall	mAP@0.5	mAP@0.5-0.95	Parameters	GFLOPS	FPS
YOLOv11n	0.859	0.832	0.876	0.673	2.6	6.3	126.6
YOLOv11n + SPD-Conv	0.864	0.837	0.882	0.695	2.2	5.4	129.6
YOLOv11n + CPCA	0.871	0.838	0.889	0.702	2.7	6.5	123.7
YOLOv11n + SDLoss	0.864	0.835	0.885	0.691	2.6	6.3	126.6
YOLOv11n + SPD-Conv + CPCA	0.877	0.841	0.896	0.718	2.3	5.6	127.9
YOLOv11n + SPD-Conv + SDLoss	0.867	0.838	0.887	0.707	2.2	5.4	129.6
YOLOv11n + SDLoss + CPCA	0.875	0.840	0.894	0.719	2.7	6.5	123.7
Ours	0.879	0.842	0.898	0.724	2.3	5.6	127.9

## Data Availability

Publicly available datasets were used in this study. These data can be found here: https://github.com/Whiffe/SCB-dataset (accessed on 31 July 2025).
